# Viral-mediated fusion of mesenchymal stem cells with cells of the infarcted heart hinders healing via decreased vascularization and immune modulation

**DOI:** 10.1038/srep20283

**Published:** 2016-02-05

**Authors:** Brian T. Freeman, Brenda M. Ogle

**Affiliations:** 1 Department of Biomedical Engineering, University of Minnesota – Twin Cities, Minneapolis, MN 55455 USA; 2 Stem Cell Institute, University of Minnesota – Twin Cities, Minneapolis, MN 55455 USA; 3 Department of Biomedical Engineering, University of Wisconsin – Madison, Madison, WI 53706 USA; 4 Masonic Cancer Center, University of Minnesota – Twin Cities, Minneapolis, MN 55455 USA; 5 Lillehei Heart Institute, University of Minnesota – Twin Cities, Minneapolis, MN 55455 USA; 6 Institute for Engineering in Medicine, University of Minnesota – Twin Cities, Minneapolis, MN 55455 USA

## Abstract

Cell fusion can occur between mesenchymal stem cells (MSCs) transplanted to improve cardiac function and cells of the recipient. The therapeutic benefit or detriment of resultant cell hybrids is unknown. Here we augment fusion of transplanted MSCs with recipient cardiac cell types via viral fusogens to determine how cardiac function is impacted. Using a Cre/LoxP-based luciferase reporter system coupled to biophotonic imaging and echocardiography, we found that augmenting fusion with the vesicular stomatitis virus glycoprotein (VSVG) increased the amount of fusion in the recipient mouse heart, but led to diminished cardiac function. Specifically, MSCs transfected with VSVG (MSC-VSVG) had the lowest mean fold increase in fractional area change (FAC) and cardiac output (CO). Although the amount of fusion detected had a strong positive correlation (Pearson) with fractional area change and cardiac output at day 7, this effect was lost by day 28. The decrease in cardiac function seen with MSC-VSVG treatment versus MSC alone or sham treatment was associated with decreased MSC retention, altered immune cell responsiveness and reduced vascularization in the heart. This outcome garners consideration in the context of cellular transplantation to damaged tissues, those with viral infection or other microenvironmental conditions that might promote fusion.

One of the most prevalent health issues in first world countries continues to be myocardial infarction^1^. Mesenchymal/multipotent stem/stromal cell (MSC) therapy has been viewed as a promising treatment to solve this issue^2^,^3^,^4^,^5^,^6^,^7^,^8^. MSCs have the ability to home to injured tissues^9^,^10^, secrete paracrine factors that allow for immune evasion^11^,^12^,^13^ and/or increase angiogenesis^10^,^14^,^15^,^16^,^17^,^18^,^19^. In the course of these studies, many have observed fusion between MSCs and cardiac cells^20^,^21^,^22^,^23^,^24^,^25^,^26^,^27^,^28^,^29^,^30^. However, the impact of cell fusion in this scenario and subsequent reprogramming on cardiac function at the cellular and tissue scale is not well understood.

Fusion of MSCs with cardiac cell types may improve cardiac function if the fusion products adopt the phenotype and associated function of cardiac cell types including cardiomyocytes, smooth muscle cells and endothelial cells. Evidence from the literature suggests stem cells and somatic cells can give rise to fusion products with characteristics of the somatic cell, thereby effectively programming the stem cells. For example, Blau *et al.* fused differentiated mouse muscle cells and human amniocytes and found that the mature cell phenotype dominated such that the amniocytes expressed human muscle proteins via exchange of cytomplasmic components^31^. Recent studies have shown that fusion of bone marrow-derived cells with hepatocytes has a therapeutic effect on the liver because the bone marrow-derived cells repopulate damaged liver tissue and adopt the biochemical functions of hepatocytes, including maintaining correct levels of serum transaminases, bilirubin and amino acids^32^,^33^,^34^,^35^.

Fusion of MSCs with cardiac cell types could also improve cardiac function if the fusion products adopt the phenotype and associated function of mesenchymal stem cells, such as self-renewal, pro-angiogenic propensity and anti-inflammatory effects. Evidence from the literature suggests fusion products of stem cells and somatic cells can serve to effectively reprogram the somatic cell to a less mature state. For example, Cowan *et al.* reverted human fibroblasts to a pluripotent-like state after fusion with embryonic stem cells^36^. Tada *et al.* observed a similar pluripotent hybrid cell after fusing embryonic germ cells and lymphocytes^37^.

Alternatively, fusion of MSCs with cardiac cell types may hinder cardiac function if the fusion products adopt a phenotype and associated function distinct from either cardiac cell types or mesenchymal stem cells. Blau *et al.* found heterokaryons formed from muscle cells and keratinocytes, expressed a combination of both gene profiles^38^. A similar result was seen after fusing intestinal epithelial cells and macrophages in a murine model of intestinal cancer in that cell fusion hybrids retained the transcriptome identity characteristic of both parental cells, but also expressed genes not activated in either parent cell type^39^. The activation of previously unexpressed genes is postulated to be responsible for the creation of cancer stem cells through fusion between tumor cells and bone marrow-derived cells^40^,^41^,^42^.

In the present study, we use a Cre/*LoxP*-based molecular approach to detect fusion of transplanted MSCs to cells of living mice and we utilize echocardiography to determine how MSC fusion affects cardiac function. Using this approach, we found that human mesenchymal stem cells delivered to the murine heart via a collagen-based patch fuse after delivery and that augmented fusion of MSCs via viral fusogen appears to have a detrimental affect on cardiac function. This negative outcome was associated with decreased MSC retention, reduced vascularization in the healthy heart and altered immune modulation 56 days after transplantation.

## Results

### Detection and Augmentation of MSC Cell Fusion *in vivo*

**Figure 1:**
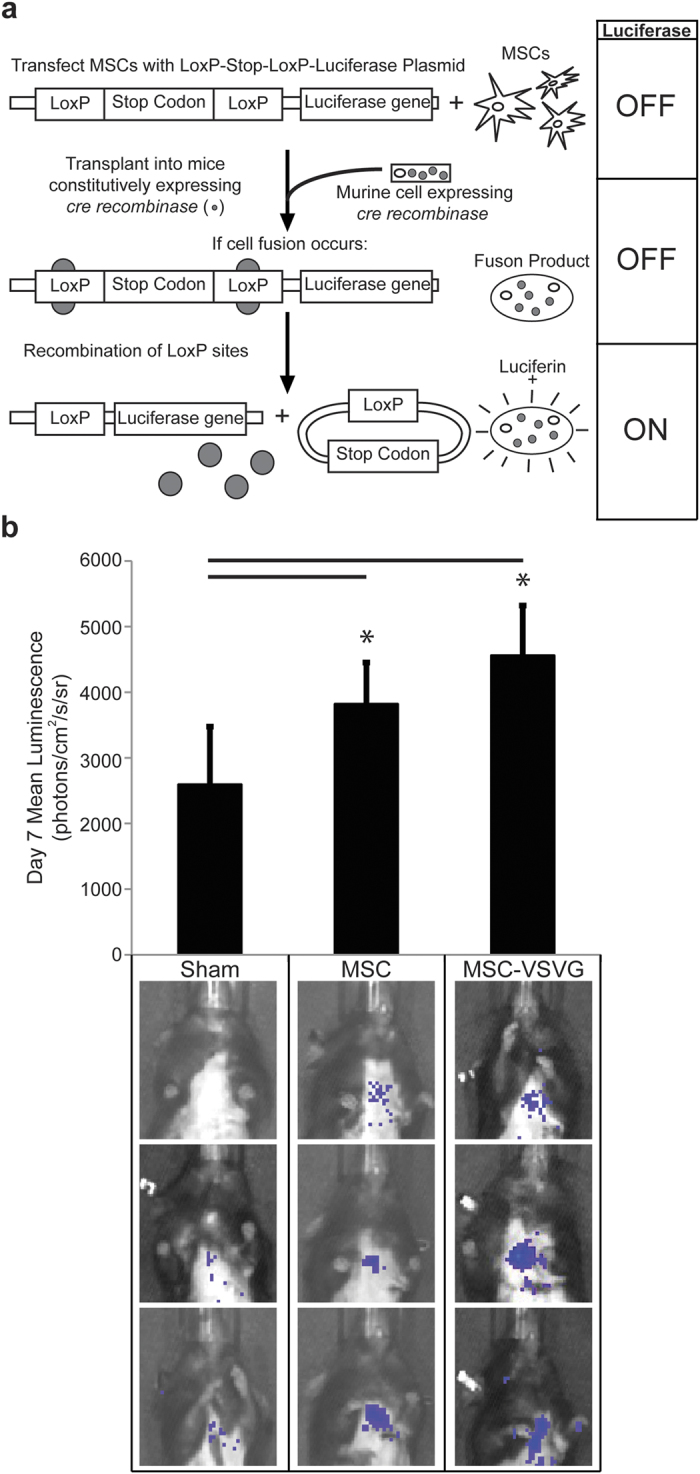
Detection and augmentation of MSC cell fusion *in vivo* (**a**) Schematic of the *in vivo* Cre/*LoxP* biophotonic detection system. MSCs are transfected with a *LoxP*-stop codon-*LoxP*-luciferase plasmid prior to cell transplantation. The MSCs are transplanted into mice, which constitutively express Cre recombinase. Upon fusion between MSCs and a cell of the Cre mouse, Cre recombinase excises the *LoxP*-stop codon-*LoxP* sequence and luciferase is expressed in the fusion product. The fusion product can then emit a bioluminescent signal after the addition of a luciferin substrate. (**b**) Quantification of the day 7 mean luminescent signal (photons/centimeters^2^/second/steradian, photons/cm^2^/s/sr) for each treatment group (sham, MSC, and MSC-VSVG). The MSC and MSC-VSVG emitted a significantly higher mean luminescent signal compared to the sham control group (*P < 0.05, data is displayed as average (Avg) + standard deviation (SD)). Three representative luminescent overlay images for each group are shown below graph.

Previously, we showed that fusion of MSCs occurs both spontaneously and with the aid of exogenously supplied viral fusogens after transplantation to the murine heart^20^,^43^,^44^. In this study, we again utilized a Cre/*LoxP*-based luciferase inducible reporter system coupled to biophotonic imaging to detect and quantify fusion between transplanted MSCs and cells of mice constitutively expressing Cre recombinase (Fig. 1a,b). The transplanted MSCs (both MSC and MSC-VSVG) were transfected with a *LoxP*-stop codon-*LoxP*–luciferase construct such that luciferase expression is limited to hybrids between donor and recipient cells (Fig. 1a). Importantly, this imaging method is non-invasive and fusion can be quantified without sacrificing the mice. Seven days following myocardial infarction and MSC cell transplantation via collagen patch (TissueMend) to the heart, bioluminescence was measured for mice with no treatment (Sham, n = 5), mice receiving MSCs with no fusogen (MSC, n = 5), and mice receiving MSCs transfected with the VSVG fusogen (MSC-VSVG, n = 5). The sham mice were used as a negative control to determine background bioluminescence (2591 + 884.9 photons per second per cm^2^ per steradian (photons/cm^2^/s/sr)). Fusion was increased in both the MSC (3818 + 762.6 photons/cm^2^/s/sr) and MSC-VSVG (4557 + 1317 photons/cm^2^/s/sr) groups compared to sham (*P < 0.05, compared to sham) (Fig. 1b). The highest amount of bioluminescent signal, and thus fusion, was observed in the MSC-VSVG (not significant compared to MSC group). These data demonstrate a trend towards enhanced fusion between MSCs and cardiac cell types via expression of viral fusogens in MSCs.

### Augmented MSC Cell Fusion Affects Function of Infarcted Myocardium

**Figure 2:**
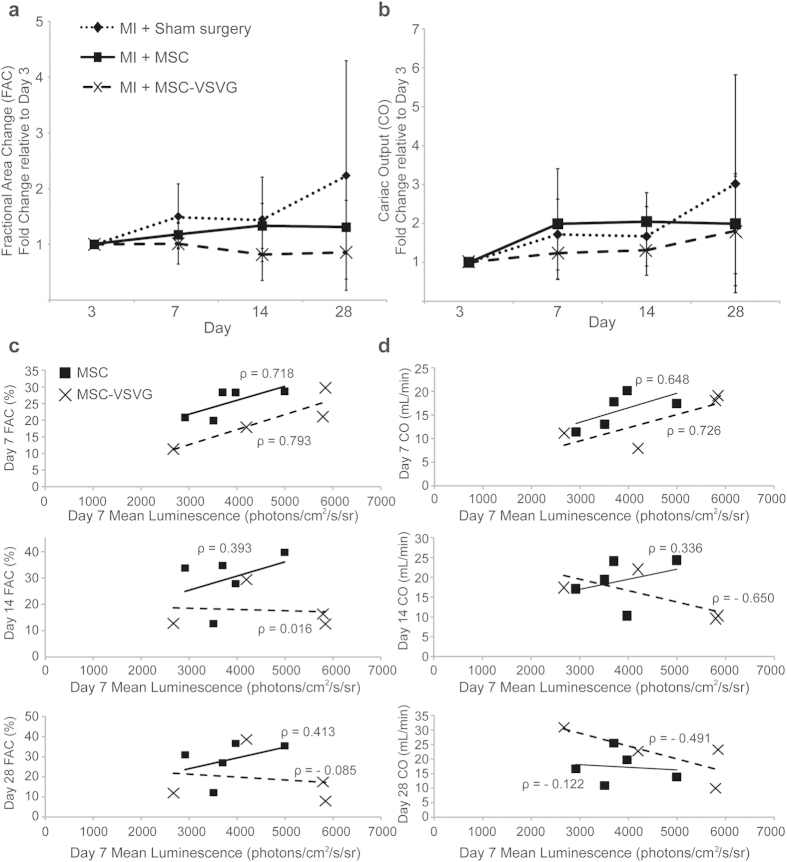
Augmented MSC cell fusion alters function of infarcted myocardium. Cardiac functional improvement is displayed relative to day 3 (per mouse) following infarction/cell delivery for (**a**) fractional area change (FAC) and (**b**) cardiac output (CO). The sham group displayed the highest fold increase over the 28 days monitored. The augmented fusion group (MSC-VSVG) showed the lowest fold change. To better probe the relationship between fusion and cardiac function, per mouse correlative analyses were conducted and are here displayed as the mean luminescent signal (i.e., amount of fusion) vs. the (**c**) FAC (%) or (**d**) CO (mL/min) for each time point. At day 7 a strong positive correlation (ρ) emerges between mean luminescent signal and cardiac function, but this correlation is lost at later time points.

To determine the impact of MSC cell fusion on cardiac function, the mice also underwent echocardiography at day 3, 7, 14 and 28 after infarction and cell delivery. Fractional area change (%) and cardiac output (mL/min) were measured at each time point for all mice of the study. The FAC and CO measurements were normalized to the day 3 time point to discern the improvement of each group (sham, MSC, MSC-VSVG) relative to the initial injury. The sham group showed the largest improvement at day 28 (2.23 + 2.06 fold increase in FAC and 3.02 + 2.80 fold increase in CO), but there was high variability from mouse to mouse (Fig. 2a,b). Interestingly, at day 28 the MSC (1.31 + 0.48 fold increase in FAC and 1.99 + 1.29 fold increase in CO) and MSC-VSVG (0.86 + 0.48 fold change in FAC and 1.81 + 1.41 fold increase in CO) groups had a lower average fold change for both FAC and CO compared to the sham group, with the augmented fusion group MSC-VSVG having the lowest relative improvement (Fig. 2a,b). These results coupled with the observed increase in fusion from the bioluminescent data of the MSC and MSC-VSVG groups (Fig. 1b) suggests that MSC fusion with cells of the mouse heart could be detrimental to the healing process in the mouse heart following myocardial infarction. However, the variability was also substantial in the MSC and MSC-VSVG groups and there were no significant differences between groups at any time point. To better probe these outcomes, a focused analysis was performed where the bioluminescent signal at day 7 for each mouse was plotted against the FAC (Fig. 2c) and CO (Fig. 2d) for each mouse at each time point. At day 7, a strong positive correlation (Pearson’s correlation coefficient) was observed between bioluminescent signal (fusion) and cardiac function (FAC and CO) for both MSC (ρ = 0.718, FAC; ρ = 0.648, CO) and MSC-VSVG (ρ = 0.793, FAC; ρ = 0.726, CO) mice. However, this trend was mostly lost over time as the positive Pearson’s correlation coefficient decreased at later time points (day 14 and day 28). The MSC group with no fusogen did exhibit a weak positive correlation through day 14, but the strength of the correlation was decreased for FAC (ρ = 0.718 at day 7 to ρ = 0.393 at day 14) and CO (ρ = 0.648 at day 7 to ρ = 0.336 at day 14). Due to the high level of variability observed between mice within the same treatment group in this study as well as the loss of correlation between fusion and function over time, the number of mice was capped at five per group so that an in depth cellular analysis could be performed to probe the mechanism behind differences in cardiac functionality.

### Human MSC Retention in Infarcted Murine Heart

**Figure 3:**
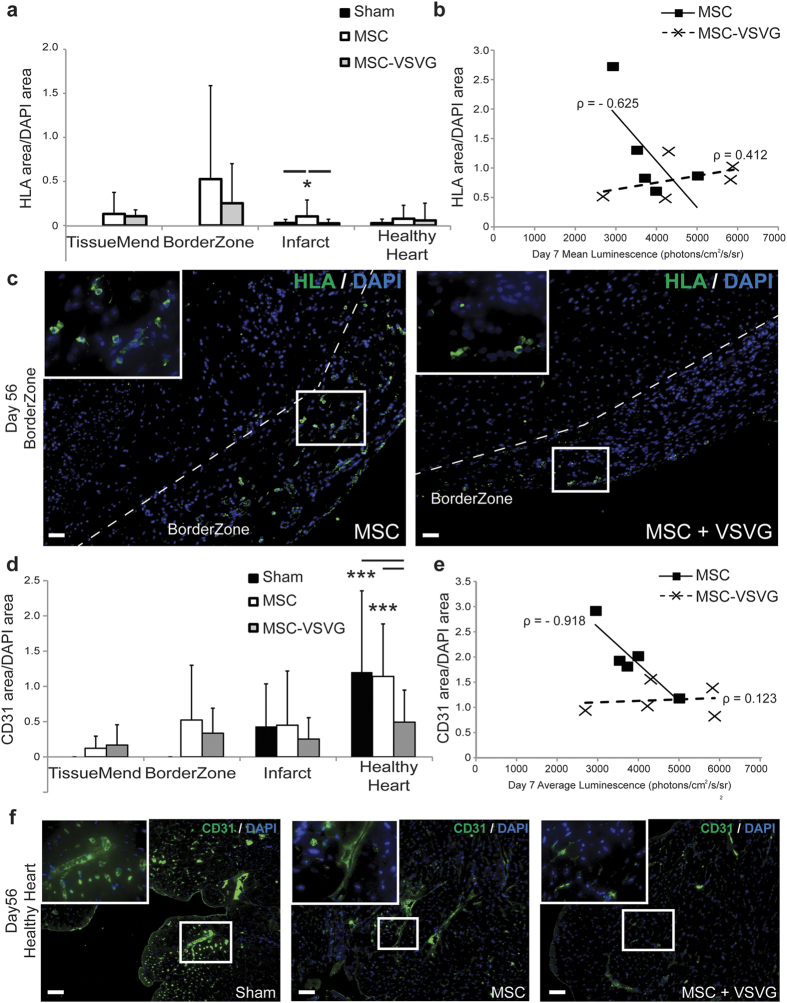
Human MSC Retention and Vascular Response in Infarcted Murine Heart (**a**) Quantification at day 56 after infarction/cell delivery of human leukocyte antigen (HLA) expression (area) normalized to DAPI signal (area) for four regions in the infarcted heart (TissueMend, Borderzone, Infarct and Healthy Heart) (5–20 images per region per sample, data displayed as Avg + SD) (*P < 0.05). (**b**) Mean luminescent signal for each mouse was plotted against the HLA area/DAPI area for each mouse. The MSC group showed a strong negative Pearson’s correlation (ρ) between mean luminescent signal (fusion) and MSC retention.(**c**) Representative images for MSC and MSC-VSVG groups in the borderzone (HLA, green and DAPI, blue) (Scale bar = 50 μm). Dashed line delineates the interface between the borderzone and the heart. (**d**) Quantification at day 56 after infarction/cell delivery of vessel density (CD31 expression area) normalized to DAPI signal (area) for four regions in the infarcted heart (5–20 images per region region per sample, data displayed as Avg + SD, ***P < 0.001). (**e**) Mean luminescent signal for each mouse was plotted against the CD31 area/DAPI area for each mouse. The MSC group showed a very strong negative correlation (ρ) between mean luminescent signal (fusion) and vessel density. (**f**) Representative images in the healthy heart (CD31, green and DAPI, blue) (Scale bar = 50 μm).

To determine whether retention of the transplanted MSCs over time played a part in the observed functional differences between MSC and MSC-VSVG groups, the hearts of the experimental mice were explanted, fixed, sectioned at day 56 and probed for human leukocyte antigen (HLA) (Fig. 3a–c) via immunofluorescence. The analysis of the heart tissues was separated into four regions (TissueMend patch, borderzone between the patch and heart, infarcted heart tissue and healthy heart tissue) to more accurately portray the spatial location in the tissue. The area of HLA positive signal was normalized to the area of DAPI signal to account for the difference in cell density in a given region. The MSC group showed the highest retention of human cells in the borderzone (0.526 + 1.06 HLA area/DAPI area), but human cells were also observed in the borderzone of the MSC-VSVG group (0.252 + 0.449 HLA area/DAPI area) albeit to a lesser extent than the MSC group (not significant) (Fig. 3a). The MSC group also showed significantly increased number of HLA positive cells in the infarcted heart (0.103 + 0.186 HLA area/DAPI area) relative to both the sham (0.028 + 0.040 HLA area/DAPI area) and MSC-VSVG group (0.025 + 0.046 HLA area/DAPI area) (*P < 0.05), even though the average HLA area/DAPI area was much lower in the infarct then the borderzone. Also of note, the MSC group showed a strong negative correlation between bioluminescence signal and HLA area/DAPI area (ρ = −0.625) (Fig. 3b). This suggests that fusion, occurring by means other than exogenously supplied VSV-G, could hinder cell retention.

### Vascular and Immune Response

In the face of similar or nearly similar retention rates of MSCs with and without augmented cell fusion, we sought to determine whether altered MSC function at the cellular level could account for differences in tissue-level function. Specifically, we probed for angiogenic stimulation and immune modulation. Vessel density in the four different heart regions was probed via CD31 expression (fluorescence area) normalized to cell number via DAPI (fluorescence area)(Fig. 3d–f). Vessel density was similar in the TissueMend, borderzone, and infarct regions between the different treatments. However in the healthy heart, the sham and MSC groups showed significantly higher CD31 area/DAPI area (1.20 + 1.15 and 1.14 + 0.744, respectively) compared to the CD31 area/DAPI area in the MSC-VSVG group (0.493 + 0.455) (***P < 0.001) (Fig. 3d). In addition, the MSC group exhibited a very strong negative correlation between bioluminescent signal and CD31 area/DAPI area (Fig. 3e) (ρ = −0.918). This trend could imply that fused MSCs lose some ability to promote angiogenesis in the infarcted heart. The healthy region of the infarcted heart typically has to compensate for the loss of contractile tissue (which would require more energy) and the increase in vessel density observed in the sham and MSC groups might represent an attempt to supply the healthy tissue with increased metabolites to match the demand due to increased workload. However, if fused MSCs lose the ability to promote angiogenesis or were reprogrammed to prevent angiongenesis, the healthy heart may have difficulty compensating for increased metabolic demand. Thus the loss of vessel density in the healthy heart region might explain, at least in part, the low-level FAC and CO improvement of the MSC-VSVG group.

**Figure 4:**
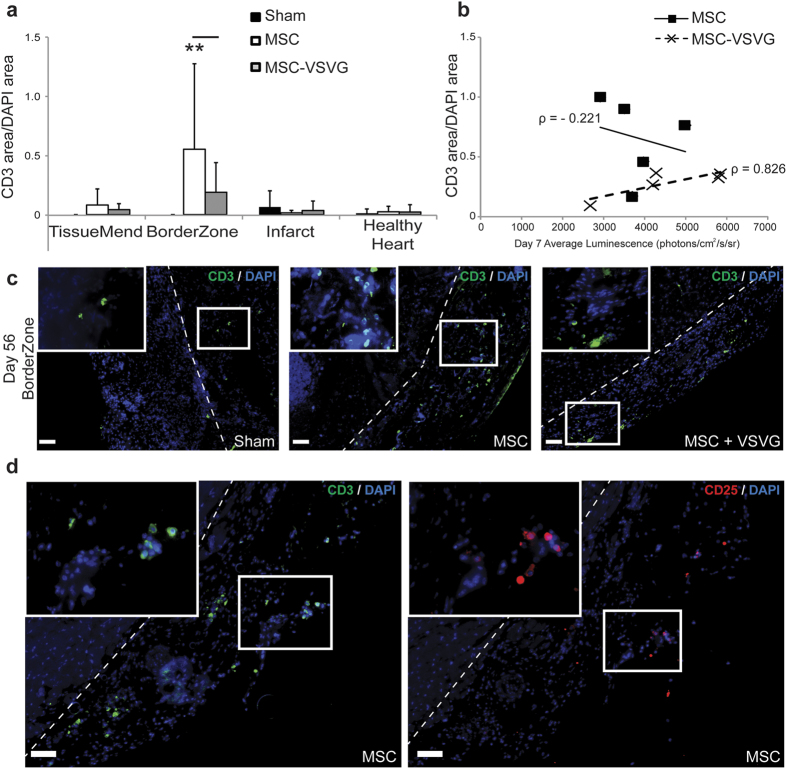
Immune modulation in the infarcted myocardium (**a**) Quantification at day 56 after infarction/cell delivery of T cell concentration (CD3 expression area) normalized to DAPI signal (area) for four regions in the infarcted heart (5–20 images per region per sample, data displayed as Avg + SD, **P < 0.005). (**b**) Mean luminescent signal for each mouse was plotted against CD3 area/DAPI area for each mouse. (**c**) Representative images in the borderzone (CD3, green and DAPI, blue) (Scale bar = 50 μm). Dashed line delineates the interface between the borderzone and the heart. (**d**) Colocalization of regulatory T cells (CD25, red) and T cells (CD3, green) seen in the borderzone of a mouse from the MSC group (DAPI, blue) (Scale bar = 50 μm). Dashed line delineates the interface between the borderzone and the heart.

Adaptive immune response was probed in the ventricles of treated and untreated mice via staining for T cells (pixel area corresponding to CD3 expression) relative to area of DAPI signal (Fig. 4a–c). Adaptive immunity was probed since the time point was far later than typical innate immune activation and since MSCs have been shown to modulate T cell function *in vitro* and *in vivo.* CD3 positive cells were rare in the sham group in all ventricle regions, as were they rare for the MSC and MSC-VSVG groups in the TissueMend, infarcted heart and healthy heart. However in the borderzone, the MSC group showed significantly more CD3 area/DAPI area (0.540 + 0.704) compared to the MSC-VSVG (0.185 + 0.244) (**P < 0.005) (Fig. 4a), with only a weak negative correlation with amount of fusion per mouse (Fig. 4b). We actually anticipated the reverse (i.e., higher T cell numbers in MSC-VSVG vs. MSC) since tissue-level function might be limited by ongoing immune activation. One potential explanation for this observation could be that all or a portion of the T cells detected in the MSC group are regulatory T cells, which suppress or downregulate induction and proliferation of effector T cells. Multiple studies have shown that human MSCs have the ability to expand regulatory T cell populations while inhibiting allostimulated T cell proliferation^45^,^46^. This could also be the reason for the higher concentration of HLA positive cells remaining the MSC group compared to the MSC-VSVG group, since the regulatory T cells might prevent clearance of the foreign human MSCs. To determine if regulatory T cells could account for all or a portion of the T cell population in the MSC group, we stained for CD25, the alpha chain of the IL-2 receptor and a known marker of regulatory T cells. CD25 positive cells were observed in the borderzone in regions that also stained positive for CD3 in MSC mice (Fig. 4d) representing approximately 33% of the CD3 positive cells. Thus decreased blood vessel density and low levels of regulatory T cells of the MSC-VSVG group with augmented fusion, suggests a potential loss of paracrine and/or immunomodulatory function of MSCs after fusing.

## Discussion

The aim of this study was to discern the affect of MSC fusion after transplantation on cardiac recovery following myocardial infarction. Three different treatment groups were utilized to help test this aim: a sham treatment, a traditional MSC cellular transplant, and a treatment wherein fusion was augmented in MSCs by the fusogen VSVG. Fusion was observed in both the MSC and MSC-VSVG group with the MSC-VSVG group showing the highest level of fusion. Unexpectedly, the sham group demonstrated the highest average fold increase in cardiac function (FAC and CO) with the augmented fusion group performing the worst for cardiac recovery after infarction. As noted earlier, fusion of MSC with parenchymal cells has been shown to aid in recovery of function in other tissues especially in the case of the liver^32^,^33^,^34^,^35^, but up to this point it was still unknown how MSC fusion in the heart might affect cardiac function. Our group and others have observed fusion of MSCs to cells of recipient heart, although most report a low level of fusion (<1% of transplanted cells) and thus the affect of the fusion on overall cardiac function was assumed to be minimal^25^,^30^. Here we report a decrease in function in the treatment group in which fusion was directly augmented. To our knowledge this is the first study to directly study the relationship between MSC fusion and cardiac function.

High variability in functional outcomes between groups, especially with fusion, led to a shift in our study design from tissue outcomes and therefore more mice (here we report five per study group) to per mouse correlative analyses and more in depth analysis of cellular-scale outcomes. This led to the separation of each mouse in the study and direct comparison of the amount of fusion detected in each mouse to the FAC and CO observed during the course of the study. Within one week of infarction and transplantation, we observed a positive correlation between the amount of fusion and cardiac functional parameters. However, this correlation was lost at later time points in the study. This suggests that perhaps MSC fusion after transplantation may have a transient positive affect due to increased cell retention at the site of injury as well as increased immune evasion due to the acquisition of mouse major histocompatibility complexes after fusion. This positive affect appears to be short lived, since the sham treatment is observed to bypass both MSC treatments at day 28. Especially in the case of the augmented fusion group (MSC-VSVG), it appears that augmenting MSC fusion hinders the ability of MSCs to promote healing. This is in line with very recent *in vitro* studies in which human MSCs, when fused with rat neonatal ventricular myocytes, downregulated sarcomeric structures and acquired a non-proliferative and non-contractile phenotype^47^. The loss of contractility and proliferation of fusion products between human MSCs and myocytes seen in this *in vitro* study helps to explain our *in vivo* observations that MSC fusion hinders improvement of fractional area change and cardiac output in the infarcted heart.

Upon observing a decrease in cardiac function associated with MSC fusion, we probed the mechanism for decreased function on the cellular level with a focus on MSC retention, vascularization, and immune modulation. A cardiac marker (such as cardiac troponin T) was not included in this study since the frequency of HLA signal in the infarct and healthy heart was extremely low. The MSC group with no fusogen had a higher average level of MSC retention in the borderzone (though not significant), higher vessel density in the healthy heart as well as a higher concentration of T cells in the borderzone than the MSC-VSVG group with the VSVG fusogen. These results could explain why the augmented fusion treatment saw reduced tissue-level function compared to the sham group. Increased fusion of MSCs via VSVG, while seeming to promote tissue function at 7 days after transplantation, appears to prevent MSCs from aiding in recovery at later time points. This could be due to the MSCs undergoing reprogramming after fusion and thus causing the MSCs to lose their innate abilities such as angiogenesis and immune modulation. The reprogrammed MSCs in the MSC-VSVG group might have lost essential paracrine capabilities, which rendered them less effective in promoting tissue repair. One might speculate that fusion “forced” via viral fusogen is artificial and therefore is not relevant *in vivo.* However, it is well documented that viral infection can facilitate fusion *in vivo*^20^,^44^,^48^,^49^,^50^,^51^,^52^,^53^,^54^. In fact, it is possible that viral infection may have been the cause of all or a portion of the “spontaneous” fusion observed in the MSC group. Indeed, the MSC group saw a negative correlation between amount of fusion and MSC retention as well as vascularization after 56 days. This spontaneous fusion seen in the MSC group also appears to negatively affect the ability of MSCs to promote healing in the infarcted myocardium. The mice in the MSC group with no fusogen that exhibited the highest amount of MSC retention and vascularization were the mice with the lowest observed fusion levels. Interestingly, the MSC group with no fusogen and low-level fusion showed an increase in CD3 positive T cells relative to the MSC-VSVG group and many of these cells were found to colocalize with CD25 positive regulatory T cells in parts the borderzone. The regulatory T cells may have enabled higher MSC retention and improved cardiac functional response seen in the MSC group.

Taken in whole, this study is the first to examine how MSC fusion after cell transplantation affects cardiac function following myocardial infarction. The negative functional impact observed, even with the small number of mice tested, should be considered for future clinical trials. MSC transplantation has been shown to be an effective treatment when cell fusion is reported at low levels. However, if cell fusion is somehow increased due to viral infection or environmental conditions after transplantation, the treatment could result in a loss of function and a negative prognosis (especially in the infarcted heart).

## Methods

### Transgenic Mice

We used transgenic mice that constitutively express Cre recombinase (B6.C-Tg[CMV-cre]1Cgn/J; Jackson Laboratory, Bar Harbor, ME) (15 total mice, 5 per treatment group, 2 months old), such that deletion of *LoxP*-flanked genes occurs in all tissues, including germ cells. The *Cre* gene is under transcriptional control of the cytomegalovirus (CMV) minimal promoter and is X-linked. The Cre sequence was introduced to BALB/cJ derived BALB/c-I embryonic stem cells (ESCs). The resulting mice were backcrossed to the BALB/c background for 8 generations and then backcrossed to the C57BL/6J background for 10 generations^55^. Only male Cre mice were used in the study owing to a false-positive signal detected when imaging the female transgenic mice (data not shown).

### Cell Culture

Human MSCs derived from human embryonic stem cells (MSCs from WA-01, a gift from Dr. Peiman Hematti, University of Wisconsin-Madison, Madison, WI. WA-01 cells were obtained via a protocol approved by the University of Wisconsin-Madison, Institutional Review Board) were expanded and cultured, as previously described^56^. In brief, MSCs were cultured on a 0.1% gelatin (Sigma-Aldrich, St. Louis, MO) pretreated flask containing α-minimum essential medium (MEM)-complete. Complete α-MEM consisted of α-MEM (Invitrogen, Carlsbad, CA), 10% fetal bovine serum (HyClone Laboratories, Logan, UT), 0.1 mM nonessential amino acids (Invitrogen), and 2 mM l-glutamine (Invitrogen). hMSC cultures were allowed to grow to 60%–70% confluence and were replated at a concentration of 1,500 cells per cm^2^. These human ESC-derived MSCs have cell surface markers, differentiation potential, and immunologic properties *in vitro* that are similar to those of adult BM-derived MSCs^56^.

### Gene Transfer

MSCs were transiently transfected with viral fusogen VSV-G^20^,^57^ to promote cell-cell fusion (MSC-VSVG) or no fusogen (MSC). In addition, MSCs in both the MSC and MSC-VSVG groups were simultaneously transfected with the luciferase gene adjacent to a floxed stop codon (p231 pCMVe-betaAc-STOP-luc; Addgene, Cambridge, MA)^23^. Transfection was accomplished using the Neon Transfection System (Invitrogen), as previously described^58^ (Supplementary Figure S1). All recombinant DNA research was conducted according to NIH guidelines and in accordance with the University of Wisconsin-Madison and University of Minnesota-Twin Cities institutional biosafety committees.

### Myocardial Infarction and Cell Delivery

Mice underwent an infarction procedure by left coronary artery ligation, such as is routinely performed at the University of Wisconsin Cardiovascular Physiology Core Facility^23^,^44^,^59^. Transfected MSCs (MSC or MSC-VSVG) were delivered to the myocardium of mice immediately after infarction via a collagen patch (TissueMend; TEI Biosciences, Boston, MA), as previously described^20^,^43^,^44^. A control (sham) was performed with only the infarction and no patch delivery. TissueMend matrices (2 × 2 × 0.8 mm) were placed in a 24-well plate (Falcon; Thermo Fisher Scientific, Pittsburgh, PA) and hydrated with α-MEM-complete culture medium. After electroporation, MSCs were seeded on the TissueMend sections at a concentration of 1 × 10^6^ cells per milliliter. The medium was changed at 24 and 48 hours, at which point the TissueMend matrix, containing ∼1 × 10^5^ transfected MSCs, was attached to the myocardium with a single suture (7-0 Prolene; Ethicon, Johnson & Johnson, New Brunswick, NJ) at each corner of the matrix. A matrix was placed such that it was in contact with both the infarct and the peri-infarct regions of the myocardium^20^,^43^,^44^. All animal procedures were performed in accordance with the guidelines of the American Association for Laboratory Animal Science and were approved by the University of Wisconsin-Madison Institutional Animal Care and Use Committee and the University of Minnesota-Twin Cities Institutional Animal Care and Use Committee.

### Bioluminescent Imaging

Recipient mice constitutively expressed Cre recombinase; therefore, when transplanted human MSCs fused with cells of the recipient, the *LoxP* sites were cleaved, and the stop signal was excised, allowing expression of luciferase. Luciferase expression was detected 7 days after cell transplantation in living mice using an *in vivo* imaging system (IVIS) (IVIS Spectrum; Caliper Life Sciences, Hopkinton, MA), as previously described^23^,^43^. The average radiance was determined by measuring the emitted photons per second per cm^2^ per steradian of the heart region using the Living Image *In Vivo* Imaging Software (PerkinElmer, Life and Analytical Sciences, Waltham, MA).

### Echocardiography

Mice underwent echocardiography 3 days post-infarction/cell delivery to obtain a baseline measurement of each mouse’s cardiac function. Further echocardiography was repeated at 7, 14 and 28 days after infarction/cell delivery to track mouse cardiac health over time. Transthoracic echocardiography was performed by using a Visual Sonics 770 ultrasonograph with a 30-MHz transducer (RMV 707B) (Visual Sonics, Toronto). Mice were lightly anesthestized with isoflurane (1%) and maintained on a heated platform. Two-dimensionally guided M-mode images of the long axis of the LV were acquired with the probe in different 3 planes, 1) sagittal plane, 2) 45% to the sagittal plane and 3) frontal plane. Images were recorded and the LV endocardial area traced at end-diastole and systole. Volumes were calculated from these areas and function expressed as fractional area change (FAC, %) and cardiac output (CO, mL/min). All parameters were measured over at least three consecutive cycles.

### Fluorescence Microscopy

Murine hearts were harvested 8 weeks after cell delivery to determine the amount of MSC retention, vascularization and immune response at the cellular level. After excision, the hearts were bisected longitudinally through the matrix. The hearts were immediately placed into 10% buffered formalin (pH 7.2; Thermo Fisher Scientific) for 24 hours, followed by 24 hours of fresh 10% buffered formalin, and a final 24-hour incubation in 70% ethanol. The samples were further processed for paraffin embedding and sectioning, as previously described^60^. For immunohistochemistry (IHC) analysis, heart sections were deparaffinized by incubating at 60 °C for 1 hour and then washed for 6 minutes in Xylene twice. The sections were rehydrated by dipping the sections 15 times each in 100% ethanol, 100% ethanol, 95% ethanol, and, finally, ultrapure water. Antigen retrieval was accomplished either by incubating the sections for 20 minutes at 37 °C in 0.5% pepsin (Thermo Fischer Scientific) in 5 mM HCl for human leukocyte antigen (HLA) (monoclonal mouse anti-HLA-A,B,C; EMR8-5; MBL International Corp., Woburn, MA) or by incubating the sections for 25 minutes at 95 °C in citrate buffer (10 mM sodium citrate (Fisher Scientific), pH 6, 0.01% Triton-X 100 (Sigma-Aldrich)) for CD31 (polyclonal rabbit anti-PECAM-1(CD31); M-185 sc-28188; Santa Cruz Biotechnology, Santa Cruz, CA), CD3 (monoclonal mouse anti-CD3; F7.2.38; Dako, Carpinteria, CA) and CD25 (monoclonal rat anti-mouse CD25; 7D4 (RUO); BD Pharmingen, San Jose, CA). The sections were removed and allowed to cool for 10 minutes at room temperature. The sections were rinsed in 1x phosphate-buffered saline (PBS) twice for 3 minutes. A 1:25 dilution of the anti-HLA-A,B,C, -CD3 or -CD25 antibodies or a 1:50 dilution of anti-CD31 was made with dilution buffer containing 5% bovine serum albumin (HyClone), 2% goat serum (MP Biomedical, Solon, OH), 1% glycine (Sigma-Aldrich), and 0.1% triton-X (MP Biomedical). Next, 40 μl of this antibody solution was placed on each tissue section overnight at 4 °C. The sections were washed with 1x PBS and incubated for 45 minutes at 4 °C with 40 μl of a 1:200 dilution of the secondary antibody (AF647 goat anti-mouse for HLA and CD3 or AF647 goat anti-rabbit for CD31; Invitrogen) in dilution buffer. The sections were washed with 1x PBS and mounted using 1,4-diazabicyclo[2.2.2]octane (Dabco)/DAPI solution composed of 5% Dabco (Sigma-Aldrich) and 0.01% DAPI (Sigma-Aldrich) in a mixture of 50% glycerol (Thermo Fischer Scientific) and 50% 2 × PBS on a microscope coverslip sealed with nail polish. Fluorescence emission was detected using an IX71 inverted deconvolution fluorescence microscope (Olympus, Center Valley, PA). The images were acquired with a 10× or 20× UPlanFluor objective (NA = 0.5), using Metamorph software (Molecular Devices, Sunnyvale, CA) and analyzed using ImageJ (Fiji; open source software, http://pacific.mpi-cbg.de/wiki/index.php/Fiji). All hearts were stained for each antibody, and 5–20 images per region were quantified for positive expression with the number depending on the size of the region in each heart. Background fluorescence was determined using a secondary antibody-only control to set a threshold for antibody detection.

### Statistical Analysis

Statistical analyses were performed using analysis of variance with Tukey’s honest significant difference post hoc test for multiple comparisons or Student’s t test for 2 independent samples; *P* < 0.05 was considered significant. Correlations that measure the linear correlation between the luminescent signal (fusion) and a second parameter were calculated using Pearson’s correlation coefficient (ρ). Data were analyzed with SigmaPlot (Systat Software Inc, San Jose, CA, http://www.systat.com).

## Additional Information

**How to cite this article**: Freeman, B. T. and Ogle, B. M. Viral-mediated fusion of mesenchymal stem cells with cells of the infarcted heart hinders healing via decreased vascularization and immune modulation. *Sci. Rep.* **6**, 20283; doi: 10.1038/srep20283 (2016).

## Supplementary Material

Supplementary Information
