# Supplementary material for: Viral-mediated fusion of mesenchymal stem cells with cells of the infarcted heart hinders healing via decreased vascularization and immune modulation
Source: Sci Rep. 2016 Feb 5;6:20283. doi: 10.1038/srep20283 (PMC4742880; doi:10.1038/srep20283)
Supplement: Supplementary Information [file srep20283-s1.pdf]

Viral-mediated fusion of mesenchymal stem cells with cells of the infarcted heart hinders healing via decreased vascularization and immune modulation

Brian T. Freeman[1,2,3] and Brenda M. Ogle, Ph.D.[1,2,3,4,5,6*]

Author Affiliation:
1. Department of Biomedical Engineering, University of Minnesota – Twin Cities, Minneapolis, MN 55455 USA
2. Stem Cell Institute, University of Minnesota – Twin Cities, Minneapolis, MN 55455 USA
3. Department of Biomedical Engineering, University of Wisconsin – Madison, Madison, WI 53706 USA
4. Masonic Cancer Center, University of Minnesota – Twin Cities, Minneapolis, MN 55455 USA
5. Lillehei Heart Institute, University of Minnesota – Twin Cities, Minneapolis, MN 55455 USA
6. Institute for Engineering in Medicine, University of Minnesota – Twin Cities, Minneapolis, MN 55455 USA

Supplementary Figures

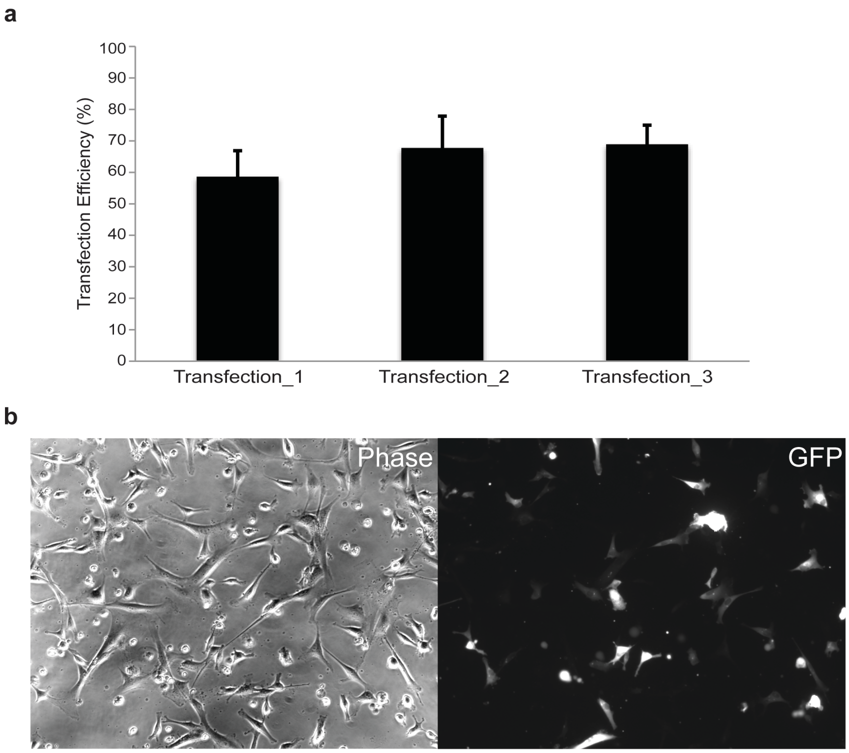

Figure S1. Transfection efficiencies associated with electroporation of human MSCs. (**a**) Three independent transfections with a pCAGS-GFP plasmid were performed in parallel with the transfections of the transplanted hMSCs. Transfection efficiency was defined as the percentage of imaged cells that express GFP relative to the total number of cells. The data is reported as the average efficiency ± standard deviation of three different fields of view for each sample. (**b**). Representative images of transfected MSCs.
